# Prevalence of and Factors Associated With Long-term Concurrent Use of Stimulants and Opioids Among Adults With Attention-Deficit/Hyperactivity Disorder

**DOI:** 10.1001/jamanetworkopen.2018.1152

**Published:** 2018-08-10

**Authors:** Yu-Jung Jenny Wei, Yanmin Zhu, Wei Liu, Regina Bussing, Almut G. Winterstein

**Affiliations:** 1 Department of Pharmaceutical Outcomes and Policy, College of Pharmacy, University of Florida, Gainesville; 2 Now with Office of Surveillance and Epidemiology, Center for Drug Evaluation and Research, US Food and Drug Administration, Silver Spring, Maryland; 3 Department of Psychiatry, College of Medicine, University of Florida, Gainesville; 4 Department of Epidemiology, College of Medicine and College of Public Health and Health Professions, University of Florida, Gainesville

## Abstract

**Importance:**

There exist limited data on the long-term concurrent use of stimulants and opioids among adults with attention-deficit/hyperactivity disorder (ADHD), a population at risk for prescription drug abuse.

**Objective:**

To assess the prevalence and secular trends of and the factors associated with long-term concurrent use of stimulants and opioids among adults with ADHD.

**Design, Setting, and Participants:**

This cross-sectional study assessed Medicaid Analytic eXtract data from 29 states between 1999 and 2010. Medicaid fee-for-service enrollees aged 20 to 64 years with ADHD who were continuously enrolled for more than 12 months after receiving an ADHD diagnosis were included. One 12-month continuous enrollment period was randomly selected as an observation unit for each enrollee. Multivariable regression models were used to determine secular trends in the prevalence of and the potential risk factors associated with long-term concurrent stimulant-opioid use. Analyses were conducted between January 1 and December 31, 2017.

**Exposures:**

Risk factors measured during the first half of the 12-month observation unit.

**Main Outcomes and Measures:**

Prevalence of long-term use of stimulants and opioids overlapping for at least 30 consecutive days was measured during the second half of the randomly selected 12-month observation unit.

**Results:**

Of the 66 406 Medicaid-enrolled adults with ADHD who were identified as eligible, 35 670 (53.7%) were 20 to 30 years old, 37 155 (56.0%) were women, and 52 551 (79.1%) were non-Hispanic white individuals. Of these 66 406 adults with ADHD, 21 723 (32.7%) used stimulants, and 3590 (5.4%) were long-term users of stimulants and opioids. Long-term opioid use was more common among adults with ADHD who used stimulants (3590 of 21 723 [16.5%]) than among those with ADHD who did not use stimulants (5826 of 44 683 [13.0%]). Long-term concurrent stimulant-opioid use increased between 1999 and 2010 (adjusted prevalence relative ratio [PRR], 1.12; 95% CI, 1.10-1.14). Compared with patients aged 20 to 30 years, the prevalence of long-term concurrent stimulant-opioid use was higher among patients in their 30s (PRR, 1.07; 95% CI, 1.07-1.08) and was further increased among patients in their 40s (PRR, 1.14; 95% CI, 1.12-1.15) and 50s (PRR, 1.17; 95% CI, 1.16-1.19). Other strongly associated risk factors included being non-Hispanic white (black PRR, 0.93; 95% CI, 0.92-0.93; other PRR, 0.97; 95% CI, 0.97-0.98; vs white), living in the southern United States (Midwest PRR, 0.98; 95% CI, 0.97-0.98; Northeast PRR, 0.94; 95% CI, 0.93-0.94; West PRR, 0.95; 95% CI, 0.94-0.96; vs South), and receiving a diagnosis of substance abuse disorder (PRR, 1.04; 95% CI, 1.03-1.05), depression (PRR, 1.02; 95% CI, 1.01-1.03), anxiety disorder (PRR, 1.05; 95% CI, 1.04-1.07), chronic pain (PRR, 1.10; 95% CI, 1.07-1.13), chronic obstructive pulmonary disease (PRR, 1.05; 95% CI, 1.04-1.07), or cardiovascular disease (PRR, 1.02; 95% CI, 1.01-1.03).

**Conclusions and Relevance:**

Long-term concurrent use of stimulants and opioids among adults with ADHD is common. This study suggests that clinical and research priorities should be made toward understanding the benefits and risks of long-term coadministration of stimulants and opioids in the management of ADHD and co-occurring pain conditions.

## Key Points

**Question:**

What are the risk factors for and prevalence of long-term concurrent stimulant and opioid prescriptions among adults with attention-deficit/hyperactivity disorder?

**Findings:**

In this multiyear, cross-sectional study of 66 406 Medicaid-enrolled adults with attention-deficit/hyperactivity disorder, long-term concurrent stimulant and opioid use was common (5.4%) and increased from 1999 to 2010. Prevalent long-term stimulant-opioid use was significantly associated with older age, non-Hispanic white race/ethnicity, southern US residency, or a pain, anxiety disorder, or substance use disorder diagnosis.

**Meaning:**

The common long-term stimulant-opioid use observed among adults with attention-deficit/hyperactivity disorder warrants further investigation to understand the association between this drug combination and patient health outcomes.

## Teaser

This cross-sectional analysis of US Medicaid files from 29 states investigates the prevalence of and risk factors associated with concurrent stimulant and opioid use among Medicaid-enrolled adults with attention-deficit/hyperactivity disorder.

## Introduction

Attention-deficit/hyperactivity disorder (ADHD) is a common neurodevelopmental disorder that typically starts in childhood.^1^ The symptoms of ADHD, such as inattention, impulsivity, disorganization, and, to a lesser extent, hyperactivity, can carry into adulthood.^1,2^ More than two-thirds of children who receive a diagnosis of ADHD continue to experience at least 1 ADHD symptom during their adult lives.^3^ Current estimates show that approximately 4% of the US adult population lives with ADHD.^4^ Persistent ADHD symptoms can adversely affect many dimensions of an individual’s life, including physical, social, occupational, and behavioral functioning and can lower overall quality of life.^5,6^

Stimulants and the nonstimulant atomoxetine are recommended as first-line treatments of ADHD.^7,8^ Although long-term use of stimulants at appropriate therapeutic doses is considered safe, combination therapy of stimulants with other drugs with euphoric effects, such as opioids, may increase the risk of drug dependence.^9^ Both stimulants and opioids can increase dopamine release in the brain—the former directly enhances the effect of dopamine, whereas the latter indirectly promotes dopamine levels by activating opioid receptors.^10,11,12^ Thus, stimulants and opioids, when used concurrently, may synergistically reinforce dopamine signals and prolong the action of dopamine neuronal activities, leading to euphoric effects.^10,11,12,13^

Despite the potential enhanced abuse risk of coadministered stimulant and opioid drugs, the United States does not restrict central nervous system stimulant prescriptions for individuals with substance use disorder or for those receiving opioid treatment, although such policy has been implemented elsewhere.^14^ Both opioids and stimulants (eg, methylphenidate, methamphetamine, or amphetamine) are regulated as controlled substances in the United States.^15,16^ Given the more than 8-fold increase in stimulant prescriptions and 4-fold increase in opioid prescriptions during the past 20 years,^17^ concerns have intensified regarding increased risk for misuse or abuse of these medications, alone or together, and the sequelae.^18,19^

The prevalence of ADHD diagnosis and treatment has dramatically increased in the past decade among adults, especially among those insured by Medicaid.^20^ With this increase and with the potential risk of prescription drug abuse among adults with ADHD, it is crucial to understand whether long-term concurrent use of stimulants and opioids is common, whether the likelihood of such a drug combination changes over time, and whether there are certain factors associated with adults with ADHD that may cause them to be more likely to use both drugs long term. The present study provides evidence of the prevalence and secular trends of and the factors associated with long-term concurrent stimulant-opioid use among adult Medicaid enrollees with ADHD.

## Methods

### Study Design and Source

This multiyear, cross-sectional study used Medicaid Analytic eXtract (MAX) files of 29 states from 1999 to 2010. The MAX files contain beneficiary billing records for inpatient and outpatient encounters and pharmacy-filled prescriptions. The inpatient and outpatient records include details on procedures and diagnoses coded using the *International Classification of Disease, Ninth Revision, Clinical Modification* (*ICD-9-CM*) billing codes. Beneficiary demographics, enrollment status, and mortality information are provided in the MAX personal summary file. This study followed the Strengthening the Reporting of Observational Studies in Epidemiology (STROBE) reporting guideline for cross-sectional studies. The University of Florida, Gainesville, and the Centers for Medicare & Medicaid Services (CMS) institutional review and privacy boards approved this study with a waiver of informed consent and a waiver of Health Insurance Portability and Accountability Act authorization.

The majority of the data were obtained before the CMS suppressed all substance use disorder–related encounter claims for its beneficiaries under the Confidentiality of Alcohol and Drug Abuse Patient Records Regulations. The regulations underwent a change in early 2017, revoking redaction of substance use disorder claims. Although we are awaiting CMS approval for a new data user agreement for more recent data (up to 2013), we used MAX data from 1999 to 2010 for the present study to provide timely reports on long-term concurrent use of stimulants and opioids among adults with ADHD. Data analyses were conducted between January 1 and December 31, 2017.

### Study Sample

To construct the study sample, we first identified adult patients enrolled in Medicaid fee-for-service plans who were aged 20 to 64 years and had at least 1 inpatient or 2 outpatient visits that were coded with an ADHD diagnosis (*ICD-9-CM* codes 314.xx) during 1999 and 2010.^21^ To address enrollment gap issues (ie, disruptions in health plan enrollment that lead to no insurance coverage or temporary employer-based coverage, which is commonly seen in Medicaid beneficiaries),^22^ we randomly selected for each patient one 12-month continuous enrollment period following receipt of an ADHD diagnosis. We used the first half of each 12-month observation period to determine baseline demographic and clinical characteristics and used the second half (ie, 6-month follow-up) to ascertain long-term concurrent use of stimulants and opioids (outcome). This approach that randomly selects an observation period for each patient has been used in previous studies to generate population-based estimates of medication exposure.^23,24^

### Medications of Interest

The medications of interest captured through the MAX pharmacy files included drug treatment of ADHD and opioids. The ADHD medications for adults included atomoxetine and stimulants (methylphenidate, dexmethylphenidate, mixed amphetamine salts, dextroamphetamine, methamphetamine, and pemoline). We included the following opioid agents approved for use in the United States by 2010: buprenorphine, butorphanol, codeine, dihydrocodeine, fentanyl, hydrocodone, hydromorphone, levorphanol, nalbuphine, meperidine, methadone, morphine, opium, oxycodone, oxymorphone, pentazocine, propoxyphene, tapentadol, and tramadol.

### Measure of Long-term Concurrent Stimulant-Opioid Use

We examined daily stimulant or opioid exposure based on the number of days’ supply of prescription claims during the 6-month follow-up of patients who received a diagnosis of ADHD. A grace period of 7 days was used to account for delayed prescription fills.^25^ Among adults with ADHD, we assessed the proportion of patients having concurrently used stimulants and opioids for at least 30 consecutive days. The minimum of 30 days was used to capture scenarios in which both stimulants and opioids appeared to be used long term, thus increasing the risk for substance use disorder and prescription drug abuse.^19^ To assess the robustness of our findings, we conducted a sensitivity analysis using a different cutoff period to define concurrent use of stimulants and opioids (ie, concurrent use for ≥15 days).

### Demographic and Clinical Characteristics

Candidate factors associated with long-term concurrent stimulant-opioid use measured at baseline included sociodemographic characteristics and mental and physical comorbidities. Sociodemographic variables included age at baseline, sex, race/ethnicity, rural vs urban residence, reasons for Medicaid eligibility, and state of residence. Age was categorized into 4 groups (20-30, 31-40, 41-50, and 51-64 years). The race/ethnicity classification was based on the information available from the MAX data. Because of the small sample size, Hispanic, Asian, Pacific Islander, and Native American individuals were classified as “other.” The 29 states of residence were categorized into 4 census regions (South, Midwest, Northeast, and West; see the classification of states in the footnote to Table 1) to ensure sufficient sample size for analysis. Mental health comorbidities included schizophrenia, bipolar disorder, depression, anxiety disorder, and substance use disorder. Physical health comorbidities included obesity, diabetes, cardiovascular disease, chronic obstructive pulmonary disease (COPD), and chronic pain, which are prevalent among patients with ADHD^26^ and represent potential factors associated with prescription opioid use.^27^ Both mental and physical comorbidities were ascertained based on the presence of at least 1 relevant diagnosis code in any position on inpatient and outpatient encounter claims (eTable 1 in the Supplement).

**Table 1.** Baseline Characteristics of Eligible Medicaid-Enrolled Adults With ADHD Between 1999 and 2010, Overall and Stratified by Stimulant Use^a^

Characteristic	Adults With ADHD, No. (%)						
	Overall Sample	No Stimulant Use (n = 44 683)			Stimulant Use (n = 21 723)		
		No Opioid Use	Short-term Opioid Use	Long-term Opioid Use	No Opioid Use	Short-term Stimulant-Opioid Use	Long-term Stimulant-Opioid Use
Sample size	66 406 (100)	27 476 (100)	11 381 (100)	5826 (100)	13 129 (100)	5004 (100)	3590 (100)
Age, mean (SD), y	31.8 (10.2)	30.1 (9.7)	31.3 (9.5)	37.6 (10.4)	30.6 (9.8)	33.1 (9.9)	38.7 (10.3)
20-30	35 670 (53.7)	16 971 (61.8)	6267 (55.1)	1645 (28.2)	7667 (58.4)	2268 (45.3)	852 (23.7)
31-40	16 479 (24.8)	5964 (21.7)	2970 (26.1)	1848 (31.7)	3008 (22.9)	1542 (30.8)	1147 (32.0)
41-50	9817 (14.8)	3095 (11.3)	1550 (13.6)	1530 (26.3)	1760 (13.4)	855 (17.1)	1027 (28.6)
51-64	4440 (6.7)	1446 (5.3)	594 (5.2)	803 (13.8)	694 (5.3)	339 (6.8)	564 (15.7)
Female	37 155 (56.0)	12 650 (46.0)	7688 (67.6)	3835 (65.8)	7122 (54.3)	3400 (68.0)	2460 (68.5)
Race/ethnicity							
Non-Hispanic white	52 551 (79.1)	19 299 (70.2)	9368 (82.3)	5047 (86.6)	11 084 (84.4)	4486 (89.7)	3267 (91.0)
Non-Hispanic black	7168 (10.8)	4517 (16.4)	1127 (9.9)	347 (6.0)	897 (6.8)	198 (4.0)	82 (2.3)
Other^b^	6687 (10.1)	3660 (13.3)	886 (7.8)	432 (7.4)	1148 (8.7)	320 (6.4)	241 (6.7)
Rural residency	19 400 (29.2)	7264 (26.4)	3748 (32.9)	2023 (34.7)	3712 (28.3)	1557 (31.1)	1096 (30.5)
Medicaid eligibility^c^							
Cash assistance	47 814 (72.0)	20 896 (76.1)	8134 (71.5)	4342 (74.5)	8757 (66.7)	3229 (64.5)	2456 (68.4)
Disability	38 205 (57.5)	18 056 (65.7)	5577 (49.0)	3385 (58.1)	7094 (54.0)	2222 (44.4)	1871 (52.1)
Region at baseline^d^							
South	22 621 (34.1)	8693 (31.6)	3943 (34.7)	2073 (35.6)	4589 (35.0)	1931 (38.6)	1392 (38.8)
Midwest	27 058 (40.8)	9941 (36.2)	4883 (42.9)	2448 (42.0)	5888 (44.9)	2282 (45.6)	1616 (45.0)
Northeast	8201 (12.4)	4996 (18.2)	888 (7.8)	443 (7.6)	1402 (10.7)	268 (5.4)	204 (5.7)
West	8526 (12.8)	3846 (14.0)	1667 (14.7)	862 (14.8)	1250 (9.5)	523 (10.5)	378 (10.5)
Year of index ADHD diagnosis							
1999	2329 (3.5)	1234 (4.5)	467 (4.1)	148 (2.5)	329 (2.5)	101 (2.0)	50 (1.4)
2000	4027 (6.1)	2163 (7.9)	761 (6.7)	334 (5.7)	492 (3.8)	173 (3.5)	104 (2.9)
2001	4220 (6.4)	2165 (7.9)	870 (7.6)	344 (5.9)	557 (4.2)	182 (3.6)	102 (2.8)
2002	4491 (6.8)	2276 (8.3)	875 (7.7)	410 (7.0)	620 (4.7)	202 (4.0)	108 (3.0)
2003	6162 (9.3)	2662 (9.7)	1190 (10.5)	565 (9.7)	1107 (8.4)	415 (8.3)	223 (6.2)
2004	7925 (11.9)	2911 (10.6)	1461 (12.8)	681 (11.7)	1744 (13.3)	729 (14.6)	399 (11.1)
2005	7393 (11.1)	2823 (10.3)	1209 (10.6)	595 (10.2)	1636 (12.5)	672 (13.4)	458 (12.8)
2006	6370 (9.6)	2554 (9.3)	1055 (9.3)	562 (9.7)	1285 (9.8)	530 (10.6)	384 (10.7)
2007	5525 (8.3)	2189 (8.0)	866 (7.6)	541 (9.3)	1161 (8.8)	409 (8.2)	359 (10.0)
2008	6002 (9.0)	2379 (8.7)	927 (8.2)	592 (10.2)	1223 (9.3)	461 (9.2)	420 (11.7)
2009	6952 (10.5)	2488 (9.1)	1083 (9.5)	628 (10.8)	1577 (12.0)	644 (12.9)	532 (14.8)
2010	5010 (7.5)	1632 (5.9)	617 (5.4)	426 (7.3)	1398 (10.7)	486 (9.7)	451 (12.6)
Mental comorbidity							
Depression	16 988 (25.6)	5578 (20.3)	3080 (27.1)	2067(35.5)	3286 (25.0)	1629 (32.6)	1348 (37.6)
Bipolar disorder	10 638 (16.0)	3889 (14.2)	1838 (16.2)	1053 (18.1)	2023 (15.4)	1059 (21.2)	776 (21.6)
Anxiety disorder	11 281 (17.0)	3394 (12.4)	2028 (17.8)	1606 (27.6)	2057 (15.7)	1086 (21.7)	1110 (30.9)
Substance use disorder	8285 (12.5)	2997 (10.9)	1606 (14.1)	1177 (20.2)	1102 (8.4)	730 (14.6)	673 (18.8)
Schizophrenia	5354 (8.1)	3066 (11.2)	809 (7.1)	406 (7.0)	690 (5.3)	228 (4.6)	155 (4.3)
Pain diagnosis							
Any pain condition	1487 (2.2)	296 (1.1)	258 (2.3)	419 (7.2)	147 (1.1)	139 (2.8)	228 (6.4)
Musculoskeletal pain	1402 (2.1)	282 (1.0)	241 (2.1)	391 (6.7)	142 (1.1)	135 (2.7)	211 (5.9)
Select physical comorbidities							
Obesity	2519 (3.8)	883 (3.2)	508 (4.5)	353 (6.1)	365 (2.8)	221 (4.4)	189 (5.3)
Diabetes	3564 (5.4)	1269 (4.6)	707 (6.2)	603 (10.4)	438 (3.3)	262 (5.2)	285 (7.9)
Cardiovascular disease	7683 (11.6)	2494 (9.1)	1335 (11.7)	1390 (23.9)	1080 (8.2)	654 (13.1)	730 (20.3)
Chronic obstructive pulmonary disease	3838 (5.8)	1050 (3.8)	840 (7.4)	765 (13.1)	423 (3.2)	331 (6.6)	429 (12.0)

Abbreviation: ADHD, attention-deficit/hyperactivity disorder.

^a^ Defined as the first 6 months of a randomly selected 12-month observation period of each patient.

^b^ Included Hispanic, Asian, Pacific Islander, and Native American individuals.

^c^ A patient may qualify for Medicaid for more than 1 reason (ie, both low income and disability).

^d^ South includes Florida, Georgia, North Carolina, South Carolina, Virginia, West Virginia, Alabama, Kentucky, Mississippi, Tennessee, Arkansas, Louisiana, and Texas; Midwest includes Illinois, Indiana, Ohio, Wisconsin, Iowa, Kansas, Minnesota, Missouri, and Nebraska; Northeast includes Massachusetts, New Jersey, and New York; and West includes Idaho, New Mexico, California, and Washington.

### Statistical Analysis

We reported the baseline characteristics of the overall study sample of adults with ADHD and of the groups with or without stimulant use during the 6-month follow-up between 1999 and 2010. None of the reported variables had missing data. Among the adults with ADHD who did or did not use stimulants, we calculated the proportions having (1) no opioid use, (2) concurrent short-term opioid use (1-29 days), and (3) concurrent long-term opioid use (≥30 days, denoted as long-term concurrent use) during the 6-month follow-up. Sensitivity analysis was performed using a shorter overlap of 15 days or more to define concurrent stimulant-opioid use. Among those who were long-term concurrent users, we further analyzed types of stimulant and opioid combinations, with opioids grouped as only short-acting opioids, only long-acting opioids, and both short- and long-acting opioids. Patients who had multiple episodes of concurrent stimulant-opioid use could contribute to more than 1 type of drug combination.

We used multivariable modified Poisson regression models to analyze risk factors (ie, independent variables) associated with long-term concurrent use of prescription stimulants and opioids (ie, dependent variable) among adults with ADHD. The independent variables included age, sex, race/ethnicity, rural residency, region, mental comorbidity (depression, bipolar disorder, anxiety disorder, substance use disorder, or schizophrenia), and physical comorbidity (pain, obesity, diabetes, cardiovascular disease, and COPD). To test secular changes in the prevalence of long-term concurrent stimulant-opioid use, we included each calendar year as a dummy variable in the models. The coefficients of these annual dummy variables represented changes in the prevalence of long-term concurrent use for a given year compared with the reference year 1999. We expressed associations as prevalence relative ratios (PRRs) and reported associated 95% CIs. All analyses were performed using SAS, version 9.4 (SAS Institute Inc). A 2-sided *P* < .05 was considered statistically significant.

## Results

We identified 66 406 eligible adult patients with at least 1 inpatient or 2 outpatient visits coded for a diagnosis of ADHD between 1999 and 2010. The final study sample and the number of individuals excluded are shown in the study flowchart (Figure). In the final sample, 35 670 individuals (53.7%) were 20 to 30 years of age, 37 155 (56.0%) were female, 52 551 (79.1%) were non-Hispanic white, 47 006 (70.8%) were nonrural residents, and 38 205 (57.5%) qualified for Medicaid based on disability. During the 6-month baseline period, 25.6% of the individuals in the sample received a diagnosis of depression, and 2.2% received a diagnosis of chronic pain conditions. Of the patients who had received a code for a diagnosis of pain, most (1402 of 1487 [94.3%]) had musculoskeletal pain (Table 1).

**Figure.**
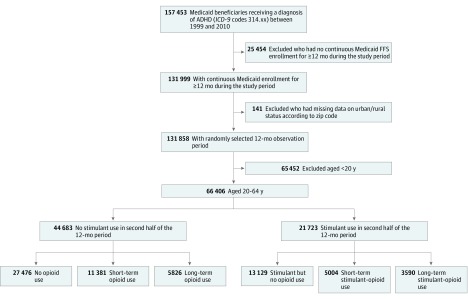
Flowchart of Patients With Attention-Deficit/Hyperactivity Disorder (ADHD) Included in the Study FFS indicates fee-for-service; *ICD-9*, *International Classification of Diseases, Ninth Revision*.

Of the 21 723 adults (32.7%) with ADHD who used stimulants during follow-up, 13 129 (60.4%) received no opioids, 5004 (23.0%) received prescription stimulants and opioids for a short term (1-29 days), and 3590 (16.5%) used these 2 classes of medications concurrently for at least 30 days. Of the 44 683 patients with ADHD who did not use stimulants, 27 476 (61.5%) received no opioids, 11 381 (25.5%) used prescription opioids for a short term, and 5826 (13.0%) used prescription opioids for a long term. Differences were noted between individuals who used opioids and individuals who did not with regard to all characteristics studied among adults with ADHD who used stimulants, as well as among those who did not use stimulants. Overall, long-term opioid use was more common among adults who used stimulants (16.5%) than among those who did not use stimulants (13.0%) (Table 1).

Among the 3590 adults with ADHD who used stimulants and opioids concurrently for a long term, 4 of every 5 adults (81.8%) used short-acting opioids, whereas only 1 of every 5 adults (20.6%) used long-acting opioids along with the stimulants. Nearly a quarter of the adults who used stimulants and opioids concurrently for a long term (23.2% [n = 832]) had prescriptions for both short- and long-acting opioids (Table 2).

**Table 2.** Combinations of Long-term Concurrent Stimulant and Opioid Use Among Adults With Attention-Deficit/Hyperactivity Disorder, 1999-2010

Drug Combination^a^	Patients, No. (%) (n = 3590)
Stimulant and short-acting opioid	2937 (81.8)
Stimulant and long-acting opioid	738 (20.6)
Stimulant, short-acting opioid, and long-acting opioid	832 (23.2)

^a^ A patient may have more than 1 type of drug combination during the 6-month follow-up. Short-acting opioids include hydrocodone, hydromorphone, morphine, oxymorphone, oxycodone, tapentadol, and tramadol in immediate-release forms; codeine and fentanyl in transmucosal and nontransdermal forms; and buprenorphine in nonpatch form. Long-acting opioids include hydromorphone, morphine, oxycodone, oxymorphone, tapentadol, and tramadol extended release, as well as buprenorphine patch, fentanyl transdermal system, levorphanol, and methadone.

We observed a significant increasing prevalence of long-term concurrent stimulant-opioid use over time (for 2010 vs 1999, adjusted PRR, 1.12; 95% CI, 1.10-1.14) (Table 3). Compared with adults in their 20s, those in their 30s showed a significantly higher prevalence of long-term concurrent stimulant-opioid use (31-40 years vs 20-30 years, adjusted PRR, 1.07; 95% CI, 1.07-1.08). Prevalence further increased as patients advanced in age (41-50 years: adjusted PRR, 1.14; 95% CI, 1.12-1.15; 51-64 years: adjusted PRR, 1.17; 95% CI, 1.16-1.19). As expected, having pain was significantly associated with long-term concurrent stimulant-opioid use among adults with ADHD (vs no pain condition, adjusted PRR, 1.10; 95% CI, 1.07-1.13). Being of non-Hispanic white race/ethnicity (black PRR, 0.93; 95% CI, 0.92-0.93; other PRR, 0.97; 95% CI, 0.97-0.98; vs white), living in the South (Midwest PRR, 0.98; 95% CI, 0.97-0.98; Northeast PRR, 0.94; 95% CI, 0.93-0.94; West PRR, 0.95; 95% CI, 0.94-0.96; vs South), and having depression (PRR, 1.02; 95% CI, 1.01-1.03), anxiety disorder (PRR, 1.05; 95% CI, 1.04-1.07), substance use disorder (PRR, 1.04; 95% CI, 1.03-1.05), COPD (PRR, 1.05; 95% CI, 1.04-1.07), or cardiovascular disease (PRR, 1.02; 95% CI, 1.01-1.03) were also factors significantly associated with long-term concurrent stimulant-opioid use, whereas receiving a diagnosis of schizophrenia appeared to be associated with protection from long-term concurrent stimulant-opioid use (adjusted PRR, 0.95; 95% CI, 0.94-0.95) (Table 3). Similar associations were found in the sensitivity analysis in which long-term concurrent stimulant-opioid use was defined as concurrent use for 15 days or more. Using the shorter cutoff to define long-term concurrent use, we found that 5541 patients with ADHD (8.3%) used stimulants and opioids concurrently (eTable 2 in the Supplement).

**Table 3.** Multivariable Modified Poisson Regression Analyses of Factors Associated With Long-term Concurrent Stimulant and Opioid Use Among Medicaid-Enrolled Adults With Attention-Deficit/Hyperactivity Disorder, 1999-2010

Variable	Long-term Concurrent Stimulant and Opioid Use (Yes vs No), PRR (95% CI)	
	Unadjusted	Adjusted^a^
Year		
1999	1 [Reference]	1 [Reference]
2000	1.01 (0.99-1.02)	1.01 (0.99-1.02)
2001	1.01 (0.99-1.02)	1.00 (0.98-1.01)
2002	1.00 (0.99-1.02)	1.00 (0.98-1.01)
2003	1.03 (1.01-1.04)	1.02 (1.00-1.03)
2004	1.06 (1.04-1.07)	1.03 (1.02-1.05)
2005	1.08 (1.06-1.09)	1.06 (1.04-1.07)
2006	1.07 (1.06-1.09)	1.06 (1.05-1.08)
2007	1.08 (1.07-1.10)	1.08 (1.06-1.10)
2008	1.09 (1.08-1.11)	1.09 (1.07-1.11)
2009	1.11 (1.09-1.12)	1.10 (1.08-1.11)
2010	1.13 (1.11-1.15)	1.12 (1.10-1.14)
Age, y		
20-30	1 [Reference]	1 [Reference]
31-40	1.09 (1.08-1.10)	1.07 (1.07-1.08)
41-50	1.15 (1.14-1.17)	1.14 (1.12-1.15)
51-64	1.20 (1.18-1.22)	1.17 (1.16-1.19)
Male (vs female as reference)	0.95 (0.95-0.96)	0.98 (0.98-0.99)
Race/ethnicity		
Non-Hispanic white	1 [Reference]	1 [Reference]
Non-Hispanic black	0.91 (0.90-0.92)	0.93 (0.92-0.93)
Other	0.95 (0.94-0.96)	0.97 (0.97-0.98)
Rural residency (yes vs no)	1.01 (1.00-1.01)	1.00 (0.99-1.01)
US Region		
South	1 [Reference]	1 [Reference]
Midwest	1.00 (0.99-1.00)	0.98 (0.97-0.98)
Northeast	0.93 (0.93-0.94)	0.94 (0.93-0.94)
West	0.97 (0.96-0.98)	0.95 (0.94-0.96)
Mental comorbidity		
Depression (yes vs no)	1.06 (1.05-1.07)	1.02 (1.01-1.03)
Bipolar didorder (yes vs no)	1.04 (1.03-1.05)	1.01 (1.00-1.02)
Anxiety disorder (yes vs no)	1.10 (1.09-1.11)	1.05 (1.04-1.07)
Substance use disorder (yes vs no)	1.06 (1.05-1.07)	1.04 (1.03-1.05)
Schizophrenia (yes vs no)	0.95 (0.94-0.96)	0.95 (0.94-0.95)
Physical comorbidity		
Pain (yes vs no)	1.18 (1.15-1.22)	1.10 (1.07-1.13)
Obesity (yes vs no)	1.04 (1.02-1.06)	1.01 (0.99-1.03)
Diabetes (yes vs no)	1.05 (1.03-1.07)	0.99 (0.97-1.00)
Cardiovascular disease (yes vs no)	1.08 (1.07-1.10)	1.02 (1.01-1.03)
COPD (yes vs no)	1.11 (1.09-1.13)	1.05 (1.04-1.07)

Abbreviations: COPD, chronic obstructive pulmonary disease; PRR, prevalence relative ratio.

^a^ The model was simultaneously adjusted for the covariates listed.

## Discussion

To our knowledge, the present study is among the first to provide population-based data on the long-term concurrent use of stimulants and opioids among adults with ADHD using Medicaid MAX files from 29 states. Our results comprising drug use data from the last decade indicated substantial and increasing long-term use of these 2 types of controlled prescription medications. Overall, 5% of adults with ADHD had concurrently used both prescription stimulants and opioids for at least 30 days during the 6-month follow-up period. The proportion was even higher among adults with ADHD who used stimulants, with 16.5% of these adults using both types of medications concurrently. The probability of long-term stimulant-opioid use compared with non–long-term use (ie, use of stimulants without opioids or short-term concurrent use of stimulants with opioids) increased by 12% between 1999 and 2010. Our findings suggest that long-term concurrent use of stimulants and opioids has become an increasingly common practice among adult patients with ADHD.

Our data also suggest that an increasing number of adults with ADHD may have developed moderate to severe chronic pain that required aggressive pain management. However, we observed only 2.2% of adults with ADHD who had received a diagnosis of any pain condition during the 6-month baseline, with the percentage higher among those with long-term opioid use (6.4% of adults who used stimulants and 7.2% of those who did not use stimulants). Our estimates are lower than self-reported data (from 18.8% to 76.7%) in surveys involving adults with ADHD,^4,28,29^ and the discrepancies may be due to differences in pain measurement, population, and time frame studied. The mechanism underlying ADHD symptoms and muscle pain is not entirely clear, but hypotheses have been proposed. Because of attention-deficit issues and increased risk-taking behavior, patients with ADHD are more prone to accidents causing physical injuries^30^ and, thereby, may be more likely to develop musculoskeletal chronic pain. Alternatively, some researchers have postulated that muscle pain is the long-term consequence of ADHD-associated motor problems (eg, difficulty in balancing, trouble with reciprocal coordination, and poor movement reflection and resistance).^31^ Given that the hypothesis involving increased pain prevalence is currently supported only by self-report survey data and small-scale clinical findings,^4,28,29,31^ formal observational studies are needed to further examine the association between ADHD and pain. An alternative hypothesis is inherent in reported associations between stimulant use and increased risk for substance use disorder,^19^ which may in turn result in increased prescription opioid use.

Although the concurrent use of stimulants and opioids may initially have been prompted by ADHD symptoms and comorbid chronic pain, continued use of opioids alone or combined with central nervous system stimulants may result in drug dependence and other adverse effects (eg, overdose) because of the high potential for abuse and misuse.^9,19,32,33^ Both opioids and stimulants are prescription drugs commonly misused by adults.^34,35,36^ National data have shown an increase in the use of these medications, and the increasing use has contributed to an increasing number of emergency department visits and overdose deaths associated with both drug classes.^9,19,32^ Of particular concern is our finding that a high proportion (81.8%) of adults who use both drugs concurrently for a long term are prescribed stimulants and short-acting opioids. Short-acting opioids are recommended for acute pain, but their long-term use has been discouraged by clinical guidelines because of the risk of abuse, opioid tolerance, and dose escalation.^37,38,39^ The common long-term use of stimulants and opioids, especially short-acting agents, observed among adults with ADHD deserves further investigation to understand the association of the use of this drug combination with patient health outcomes.

Our study also showed several important social and clinical factors associated with long-term concurrent stimulant-opioid use among adults with ADHD. We noted a significant increase in stimulant-opioid use with increasing age, even after adjusting for aging-related chronic health conditions. Our finding is consistent with a recent study of any prescription opioid use, with a reported prevalence of 8.1% among adults older than 40 years compared with a prevalence of 4.7% among adults aged 20 to 39 years.^40^ These prevalence estimations from the general adult population, which did not require a minimum 30 days’ supply, were much lower than those of long-term opioid use observed among adults with ADHD (16.5% of those who used stimulants and 13.0% of those who did not use stimulants) in the present study. Furthermore, our findings of higher long-term concurrent stimulant-opioid use among non-Hispanic white adults compared with non-Hispanic black adults and among residents from the South compared with all other US regions are consistent with previous studies on prescription opioid use.^40^

We also observed that adults with ADHD and comorbid depression, anxiety disorder, substance use disorder, cardiovascular disease, or COPD tended to use stimulants and opioids concurrently. Depression, anxiety disorder, and substance abuse disorder are strongly associated with long-term chronic opioid use,^41,42,43,44^ which may explain the increase in long-term stimulant-opioid use among adults with ADHD who have these comorbid conditions. Patients with cardiovascular disease (vs without) are more likely to experience pain,^45^ and patients with COPD symptoms are at high risk for pain and for nicotine dependence, the latter of which is an independent risk factor for substance use.^46^ Our study contributes to the understanding of the potential risk factors associated with long-term concurrent stimulant-opioid use among adults with ADHD. Identifying these high-risk patients allows for early intervention and may reduce the number of adverse events associated with the long-term use of these medications.

### Strengths and Limitations

Several strengths of this study are noteworthy. Our study provided important information on the treatment of adults with ADHD, a population that is increasing but has received limited research attention. Using more than a decade of administrative data that include detailed prescription information from 29 states allowed us to examine secular changes in concurrent stimulant-opioid use. We also explored a broad range of potentially associated factors, such as physical comorbidities, which have rarely been considered in the literature for stimulant-opioid polypharmacy.

The present study shares limitations common to observational studies based on administrative data. First, MAX data captured only prescription medications filled and reimbursed by Medicaid, without information on medications obtained through other channels, such as paying for prescription fills with cash or obtaining medications through illicit sources. Considering that opioid prescription fills are commonly paid out of pocket,^47^ our reported prevalence of concurrent stimulant-opioid use may be too low. In addition, the data provided no information on whether prescribed opioids were intended for regular use or as needed. Using strict definitions of overlapping treatment periods that were defined based on the dispensed days’ supply (assuming daily use) might have underestimated long-term concurrent use. Furthermore, our data did not allow us to determine the intended clinical indications for each prescribed medication, which limited our ability to assess the appropriateness of the observed concurrent use of stimulants and opioids. We identified the study sample of patients with ADHD based on the disease diagnosis codes for inpatient or outpatient encounters, which may have excluded patients with ADHD who did not seek medical care. Our findings from the 1999 to 2010 MAX data may not reflect recent changes in clinical opioid prescribing practice because several initiatives were implemented after 2010 to educate physicians to reduce unsafe opioid prescribing. Our study results are only generalizable to the Medicaid fee-for-service plans of 29 states. We selected these states based on absolute Medicaid enrollment numbers, resulting in the capture of more than 80% of all Medicaid beneficiaries. However, restrictions to fee-for-service plans resulted in varying representation of individual states and study years (because of growing managed care penetration over time). Future studies that extend to other states’ populations of adults with ADHD in more recent time periods will contribute to our understanding of the differences in rates and patterns of concurrent stimulant-opioid use as well as to the association of concurrent use with adverse outcomes.

## Conclusions

Among Medicaid-enrolled adults with ADHD, long-term concurrent use of stimulants and opioids was common and increased over time. Prevalent long-term stimulant-opioid use was associated with older age, non-Hispanic white race/ethnicity, southern US residency, and a diagnosis of substance abuse disorder, depression, anxiety disorder, chronic pain, COPD, or cardiovascular disease. Clinical and research priorities should be made toward understanding the benefits and risks of the long-term coadministration of stimulants and opioids in the management of ADHD and co-occurring pain conditions.

## Notes

Supplement. **eTable 1.** ICD-9 Codes for Studied Physical and Mental Comorbidities **eTable 2.** Multivariable Modified Poisson Regression Analyses of Factors Associated With Concurrent Stimulant-Opioid Use for ≥15 Days in Medicaid Adult Patients With ADHD, 29 States, 1999-2010
