# Supplementary material for: Prevalence of and Factors Associated With Long-term Concurrent Use of Stimulants and Opioids Among Adults With Attention-Deficit/Hyperactivity Disorder
Source: JAMA Netw Open. 2018 Aug 10;1(4):e181152. doi: 10.1001/jamanetworkopen.2018.1152 (PMC6324259; doi:10.1001/jamanetworkopen.2018.1152)
Supplement: Supplement. — eTable 1. ICD-9 Codes for Studied Physical and Mental Comorbidities eTable 2. Multivariable Modified Poisson Regression Analyses of Factors Associated With Concurrent Stimulant-Opioid Use for ≥15 Days in Medicaid Adult Patients With ADHD, 29 States, 1999-2010 [file jamanetwopen-1-e181152-s001.pdf]

Supplementary Online Content

Wei Y-JJ, Zhu Y, Liu W, Bussing R, Winterstein AG. Prevalence of and factors associated with long-term concurrent use of stimulants and opioids among adults with attention-deficit/hyperactivity disorder. *JAMA Netw Open*. 2018;1(4):e181152. doi:10.1001/jamanetworkopen.2018.1152

eTable 1. ICD-9 Codes for Studied Physical and Mental Comorbidities
eTable 2. Multivariable Modified Poisson Regression Analyses of Factors Associated With Concurrent Stimulant-Opioid Use for ≥15 Days in Medicaid Adult Patients With ADHD, 29 States, 1999-2010

This supplementary material has been provided by the authors to give readers additional information about their work.

© 2018 Wei Y-JJ et al. *JAMA Network Open*.

eTable 1. ICD-9 Codes for Studied Physical and Mental Comorbidities

Comorbidities	ICD-9-CM codes
**Physical disorder**	
Cardiovascular disease	390, 391, 392, 393, 394, 395, 396, 397, 398, 401, 402, 403, 404, 405, 410, 411, 412, 413, 414, 425, 426, 427, 428, 429
Diabetes	249, 250, 357.2, 362.0, 366.41
Obesity	278.0, V85.3, V85.4
Chronic Obstructive Pulmonary Disease	490, 491.0, 491.1, 491.20, 491.21, 491.22, 491.8, 491.9, 492.0, 492.8, 494.0, 494.1, 496
Pain	
Musculoskeletal pain	
Back pain	720.0-724.9
Arthritis (including fibromyalgia)	710.0-739.9
Migraines	346.0-346.9
Chronic headache	339.02, 339.12, 339.22. 339.42, 784.0, and 307.81
Neuropathic pain	250.6, 355.0, 355.9, 356.0, 357.2, and 357.9
Idiopathic pain	338.0, 338.2, 338.4
**Mental disorders**	
Depression	296.2, 296.3, 296.82, 300.4, 311
Bipolar disorder	296.0, 296.1, 296.4, 296.5, 296.7, 296.9, 296.80, 296.81, 296.89, 301.13
Anxiety disorder	300.0, 300.2, 300.3, 308.0, 308.2-308.9, 309.21, 309.81, 313.0, 313.2
Schizophrenia	295, 297, 298
Substance use disorder	291, 292, 303-305

© 2018 Wei Y-JJ et al. *JAMA Network Open*.

eTable 2. Multivariable Modified Poisson Regression Analyses of Factors Associated With Concurrent Stimulant-Opioid Use for ≥15 Days in Medicaid Adult Patients With ADHD, 29 States, 1999-2010

	Chronic concomitant stimulant-opioid use for ≥15 days		Chronic concomitant stimulant-opioid use (Yes vs. No)	
Factors	Yes (n=5,541) (%)	No (n=60,865) (%)	Prevalence relative ratio (95% confidence interval)	
			Unadjusted	Adjusted
Year				
1999	83 (1.5)	2,246 (3.7)	1 [Reference]	1 [Reference]
2000	165 (3.0)	3,862 (6.4)	1.01 (0.99, 1.03)	1.01 (0.99, 1.03)
2001	177 (3.2)	4,043(6.6)	1.01 (0.99, 1.03)	1.00 (0.99, 1.02)
2002	176 (3.2)	4,315 (7.1)	1.01 (0.99, 1.02)	1.00 (0.98, 1.01)
2003	395 (7.1)	5, 767 (9.5)	1.05 (1.03, 1.07)	1.04 (1.02, 1.05)
2004	689 (12.4)	7,236 (11.9)	1.10 (1.08, 1.12)	1.07 (1.05, 1.09)
2005	724 (13.1)	6,669 (11.0)	1.12 (1.10, 1.14)	1.09 (1.07, 1.11)
2006	590 (10.7)	5,780 (9.5)	1.11 (1.10, 1.14)	1.09 (1.07, 1.11)
2007	530 (9.6)	4,995 (8.2)	1.11 (1.09, 1.13)	1.11 (1.08, 1.13)
2008	598 (10.8)	5,404 (8.9)	1.12 (1.10, 1.14)	1.11 (1.09, 1.13)
2009	783 (14.1)	6,169 (10.1)	1.14 (1.12, 1.17)	1.13 (1.11, 1.15)
2010	631 (11.4)	4,379 (7.2)	1.17 (1.15, 1.19)	1.16 (1.13, 1.18)
Age				
18-25	1,584 (28.6)	34,086 (56.0)	1 [Reference]	1 [Reference]
26-35	1,781 (32.1)	14,698 (24.2)	1.12 (1.11, 1.13)	1.10 (1.09, 1.11)
36-45	1,442 (26.0)	8,375 (13.8)	1.19 (1.17, 1.20)	1.16 (1.15, 1.17)
46-64	734 (13.3)	3,706 (6.1)	1.22 (1.20, 1.24)	1.19 (1.17, 1.21)
Sex				
Female	3,813 (68.8)	33,342 (54.8)	1 [Reference]	1 [Reference]
Male	1,728 (31.2)	27,523 (45.2)	0.93 (0.92, 0.93)	0.97 (0.96, 0.97)

© 2018 Wei Y-JJ et al. *JAMA Network Open*.

Race/ethnicities				
Non-Hispanic White	5,047 (91.1)	47,504 (78.1)	1 [Reference]	1 [Reference]
Non-Hispanic Black	144 (2.6)	7,024 (11.5)	0.87 (0.87, 0.88)	0.90 (0.89, 0.90)
Other	350 (6.3)	6,337 (10.4)	0.93 (0.92, 0.94)	0.96 (0.95, 0.97)
Rural residency				
No	3,841 (69.3)	43,165 (70.9)	1 [Reference]	1 [Reference]
Yes	1,700 (30.7)	17,700 (29.1)	1.01 (1.00, 1.02)	1.00 (0.99, 1.01)
Region				
South	2,146 (38.7)	20,475 (33.6)	1 [Reference]	1 [Reference]
Midwest	2,488 (44.9)	24,570 (40.4)	0.90 (0.90, 0.91)	0.97 (0.96, 0.98)
Northeast	309 (5.6)	7,892 (13.0)	0.96 (0.95, 0.97)	0.91 (0.90, 0.92)
West	598 (10.8)	7,928 (13.0)	1.00 (0.99, 1.00)	0.93 (0.92, 0.94)
**Mental comorbidities**				
Depression				
No	3,501 (63.2)	45,817 (75.4)	1 [Reference]	1 [Reference]
Yes	2,040 (36.8)	14,948 (24.6)	1.09 (1.08, 1.10)	1.03 (1.02, 1.04)
Bipolar				
No	4,305 (77.7)	51,463 (84.6)	1 [Reference]	1 [Reference]
Yes	1,236 (22.3)	9,402 (15.5)	1.07 (1.06, 1.08)	1.03 (1.01, 1.04)
Anxiety				
No	3,956 (71.4)	51,169 (84.1)	1 [Reference]	1 [Reference]
Yes	1,585 (28.6)	9,696 (15.9)	1.12 (1.11, 1.13)	1.06 (1.05, 1.07)
Substance use disorder				
No	4,515 (81.5)	53,606 (88.1)	1 [Reference]	1 [Reference]
Yes	1,026 (18.5)	7,259 (11.9)	1.08 (1.07, 1.09)	1.06 (1.04, 1.07)
Schizophrenia				

© 2018 Wei Y-JJ et al. *JAMA Network Open*.

No	5,290 (95.5)	55,762 (91.6)	1 [Reference]	1 [Reference]
Yes	251 (4.5)	5,103 (8.4)	0.93 (0.92, 0.94)	0.93 (0.92, 0.94)
**Physical comorbidities**				
Pain				
No	5,236 (94.5)	59,683 (98.1)	1 [Reference]	1 [Reference]
Yes	305 (5.5)	1,182 (1.9)	1.21 (1.18, 1.25)	1.10 (1.06, 1.13)
Obesity				
No	5,246 (94.7)	58,641 (96.4)	1 [Reference]	1 [Reference]
Yes	295 (5.3)	2,224 (3.7)	1.06 (1.04, 1.08)	1.02 (1.00, 1.04)
Diabetes				
No	5,135 (92.7)	57,707 (94.8)	1 [Reference]	1 [Reference]
Yes	406 (7.3)	3,158 (5.2)	1.06 (1.04, 1.07)	0.98 (0.97, 1.00)
Cardiovascular disease				
No	4,477 (80.8)	54,246 (89.1)	1 [Reference]	1 [Reference]
Yes	1,064 (19.2)	6,619 (10.9)	1.11 (1.09, 1.12)	1.03 (1.02, 1.05)
Chronic Obstructive Pulmonary Disease				
No	4,956 (89.4)	57,612 (94.7)	1 [Reference]	1 [Reference]
Yes	585 (10.6)	3,253 (5.3)	1.13 (1.11, 1.15)	1.05 (1.03, 1.07)

© 2018 Wei Y-JJ et al. *JAMA Network Open*.
